# Association of Adiposity Indices with Hypertension in Middle-Aged and Elderly Thai Population: National Health Examination Survey 2009 (NHES-IV)

**DOI:** 10.3390/jcdd6010013

**Published:** 2019-03-13

**Authors:** Hung Nguyen Ngoc, Wantanee Kriengsinyos, Nipa Rojroongwasinkul, Wichai Aekplakorn

**Affiliations:** 1 Institute of Nutrition, Mahidol University, Nakhon Pathom 73170, Thailand; nghung9010@gmail.com (H.N.N.); nipa.roj@mahidol.ac.th (N.R.); 2 Faculty of Medicine Ramathibodi Hospital, Mahidol University, Bangkok 10400, Thailand; wichai.aek@mahidol.ac.th

**Keywords:** adiposity indices, hypertension, lipid accumulation product, waist-to-height ratio, waist circumference, conicity index, visceral fat, total body-fat

## Abstract

Obesity in terms of excess fat mass is associated with increased morbidity, disability and mortality due to obesity-related disorders, including hypertension. Many hypertensive individuals are overweight and often receive their advice to lose weight related to body-fat, in order to lower their blood pressure. However, it is still unclear whether there is a strong association of adipose tissue measured by adiposity indicators with hypertension in the Thai population. Various adiposity indices have been published to distinguish the distribution of body fat with disparate properties. This study examined nine adiposity markers and their association with hypertension in 15,842 Thai adults ≥35 years old. Data were obtained from the nationwide Thai National Health Examination Survey 2009. Accuracy performance and associations of indexes with hypertension were analyzed by Area Under Curve (AUC) and logistic regression analyses. Regardless of gender, the best methods to distinguish performance were waist-to-height ratio (WHtR) [AUC: 0.640 (0.631–0.649)], followed by lipid accumulation product (LAP) [AUC: 0.636 (0.627–0.645)], waist circumference (WC) [AUC: 0.633 (0.624–0.641)], and Conicity index (C-Index) [AUC: 0.630 (0.621–0.639)]. Linear regression analysis exhibited the independent association of the top four indices, WC, WHtR, C-Index, and LAP with higher systolic and diastolic blood pressure. Those indices’ quartiles were graded in a dose-response manner which significantly increased at the higher quartiles. The indicator’s cutoff point carried the odds ratio of presence hypertension in the range of 1.7 to 2.5 (*p* < 0.001). Among the nine obesity indices, WHtR (cutoff >0.52) in both genders was the simplest and most practical measurement for adiposity in association with hypertension in middle-aged and elderly Thais.

## 1. Introduction

Currently, obesity adversely affects populations in both developed and developing countries. For Thailand, urbanization is rapidly increasing along with accelerated socio-economic growth, which has led to over 52% of Thais now living in urban areas. Urban Thais tend to have less healthy diets and less physical activity with a more sedentary lifestyle than persons living in rural areas, which is a critical risk factor for developing obesity [1]. Obesity in terms of excess body-fat mass is strongly associated with an increase of morbidity, disability and pre-mature mortality due to the obesity-related disorders, including hypertension [2]. A previous study revealed that Thai adults of both genders and aged over 35 tend to become overweight and obese [3], with significant increases in hypertension and cardiovascular diseases compared to younger age groups [4,5]. The prevalence of obesity in Thai middle-aged persons and the elderly is continuously increasing and accounted for 48% in 2004 [6].

Various anthropometric indices have been developed and used to measure body fat composition. Body mass index (BMI) is the most widely used to assess obesity, but it is criticized for its single estimate that cannot describe the distribution of truncal adipose tissue and muscle mass nor the complex physio-pathology of excess adiposity [7]. Consequently, other alternative anthropometric indices are used to assess abdominal adipose accumulation. Waist circumference (WC), waist-to-hip ratio (WHR), waist-to-height ratio (WHtR), Conicity Index (C-Index), and the Body adiposity index (BAI) have been introduced and are strongly associated with central obesity [8,9,10,11]. Nevertheless, these indices cannot fully distinguish truncal fat compartments, including visceral fat and subcutaneous fat, while hypertension risk, metabolic risk and other causes of cardiovascular morbidity and mortality are deleteriously influenced by visceral adipose tissue rather than subcutaneous fat [12,13,14]. Consequently, measurements of visceral adipose tissue (VAT) or subcutaneous adipose tissue (SAT) are relevant and crucial in examining the relationship between obesity and high blood pressure. Several non-traditional and cutting-edge anthropometric indices have been developed and studied to solve these limitations. Visceral Adiposity Index (VAI), Lipid Accumulation Product (LAP), and Cardiometabolic Index (CMI) have been found to beneficially discriminate visceral fat from subcutaneous fat [15,16,17,18].

Many previous studies affirmed that the excess of abdominal fat is an independent predictor of a high blood pressure condition [2]. Therefore, the majority of health campaigns have focused on advising overweight and obese individuals to lose weight in order to reduce body-fat in hypertension prevention and treatment. However, it is still unclear what the extent of association of adiposity indicators with hypertension in Thai population is, especially among adults aged over 35. Therefore, this study’s objective was to investigate the association of different anthropometric and adiposity indices with hypertension among middle-aged and elderly Thai population. Nine obesity indices were examined in this study, including, BMI, WC, WHR, WHtR, C-Index, BAI, VAI, LAP, and CMI.

## 2. Materials and Methods

### 2.1. Study Population

Data were obtained from the nationwide Thai National Health Examination Survey 2009 (NHES-IV) which was approved by the Ethical Review Committee for Research in Human Subjects, Ministry of Public Health. The NHES-IV design and sampling method has been described extensively in a prior publication [19]. Briefly, the national study was based on a multistage probability cluster sampling technique where sampling units in each of the four stages were (1) five provinces in each of the four main regions (north, northeast, central, and south) and Bangkok; (2) two to three districts in each selected province; (3) 13–14 electoral units (EU)/villages in each district; and (4) individuals among six age groups (15–29, 30–44, 45–59, 60–69, 70–79, and ≥80 years) of each gender from each EU/village. The final sample size comprised 20,426 individuals and the response rate was 93.1%. The present study analyzed data for the middle-aged and elderly Thai population, which totaled 15,842 individuals aged 35 and over, of which 7506 were men and 8336 were women. A complete set of demographic and biochemical parameters were collected from all eligible participants. Pregnant women were excluded.

### 2.2. Demographic and Biochemical Data Collection

A prior report described the NHES-IV data collection procedures in detail [19]. Data included in the survey included demographic data (i.e., age, education level, the area of residence, geographic region); behavioral risk factors, such as smoking and alcohol consumption; and physical activity patterns. Anthropometric data in terms of body weight, height, waist circumference (WC), hip circumference (HC), and blood pressure were recorded using standard procedures. Participants rested in a sitting position for at least 5 min before measurement. Blood pressure was standardized and defined as systolic (SBP) and diastolic blood pressure (DBP) in mmHg. The pressure wave transmitted along the arteries with the highest pressure (SBP) is created by the heart contracting, and the lowest pressure (DBP) is measured as the heart fills. These values were measured using an automatic blood pressure monitoring device (Microlife model A100, Microlife AG, Widnau, Switzerland) with each participant’s arm being supported at the level of the heart with an appropriate arm cuff. Three blood pressure measurements were obtained from all participants and the mean readings of the second and third measurements were recorded and used for the present analysis. Weight was measured with participants wearing light clothing and barefoot to the nearest 100 g using a calibrated digital scale (TANITA model HD316, Tanita Corporation, Tokyo, Japan). Height was measured using a stadiometer to the nearest 0.1 cm. During measurement, participants stood barefoot with their shoulders in a normal position. Waist circumference was measured at a horizontal plane midway between the iliac crest and lower rib margin in centimeters to the nearest 0.1 cm. Hip circumference was measured to the nearest 0.1 cm around the thighs, at the height of the greater trochanter, in a standing position. Laboratory blood data were analyzed by fasting antecubital vein blood specimens, which were obtained from participants who had fasted for 12 h overnight before blood drawing. Triglyceride (TG), high-density lipoprotein cholesterol (HDL-C), low-density lipoprotein cholesterol (LDL-C), and fasting blood glucose (FBG) were collected, frozen and transferred to the central laboratory center at the Faculty of Medicine, Ramathibodi Hospital. FBG was analyzed by the hexokinase enzyme method. TG was measured by enzymatic colorimetric methods, while HDL-C and LDL-C were analyzed by homogeneous enzymatic colorimetric methods using the Hitachi 917 biochemistry analyzer (Roche Diagnostics, Basel, Switzerland).

### 2.3. Definitions

In the present study, individuals were diagnosed with hypertension when they had a high blood pressure of SBP ≥140 or DBP ≥90 mm Hg or used antihypertensive medication during the past 2 weeks, without any occurrence of heart failure, myocardial infarction, or stroke in the past 6 months and other relevant chronic diseases [20].

BMI was calculated according to Quetelet’s formula as body weight (kg) divided by height squared (m2); WHR and WHtR were calculated by dividing waist circumference (cm) with hip circumference and height (cm) respectively; Conicity Index (C-Index) was calculated using the equation: C-Index = WC (m)/[0.109 × √ {weight (kg)/Height (m)}] where 0.109 is a constant that results from the conversion of units of volume and mass into units of length [10]; BAI was calculated using hip circumference and height by the following formula [11]: BAI = {[hip circumference (cm)/height (m)^1.5^] −18}; VAI, a gender-specific index based on WC, BMI, TG and HDL-c, was calculated as follows: [16] Males: VAI = {WC (cm)/[39.68 + (1.88 × BMI)]} × (TG/1.03) × (1.31/HDL-C); Females: VAI = {WC (cm)/[39.58 + (1.89 × BMI)]} × (TG/0.81) × (1.52/HDL-C) where both TG and HDL-C values were expressed in mM; LAP was calculatedas: [WC (cm) −65] × [TG (mM)] for men, [WC (cm) −58] × [TG (mM)] and for women [17]; Cardiometabolic index (CMI) is calculated using the equation suggested by Ichiro Wakabayashi et al as: TG/HDL-C × WHtR [18].

### 2.4. Statistical Analysis

All statistical analyses were conducted using IBM-SPSS Statistic for Windows, version 24.0 (IBM Corp., Armonk, NY, USA). Descriptive statistics for all covariates were gender-specifically stratified by hypertension and summarized as mean (±standard deviation) or median (interquartile range) continuous variables and frequency (percentage) for categorical variables when appropriate. In univariate analysis, demographic, biochemical, and clinical characteristics between normal blood pressure subjects and those with hypertension were analyzed using an Independent *t*-test to compare mean values and the chi-squared test to evaluate differences in prevalence rates. Mann–Whitney *U* test was examined in comparing the median of non-normal distribution data by using the nonparametric test. The prevalence of hypertension was examined for linear regression analysis by quartiles of WC, WHtR, C-Index, and LAP using the chi-square test. Linear regression models were adopted to predict the changes in SBP and DBP in two models. The value of unstandardized (B) with 95% confidence interval (CI) and standardized (β) coefficient are expressed per 1 SD increment in WC, WHtR, C-Index, and LAP. In multiple regression analysis, the logistic models examined the association of WC, WHtR, C-Index, and LAP with prevalent hypertension in terms of odds ratio (OR) as categorized into quartiles, cut-off point and per 1 SD incensement of the variable; followed by the multivariable model adjusted for age (model 1); and further adjusted for living area, education background, cigarette smoking within 12 months and regular smoking, alcohol consumption, alcohol consumption level, and physical activity (model 2). A third model additionally adjusted model 2 for a log of fasting plasma glucose (FBG), HDL-C and LDL-C level. Moreover, multivariable-adjusted ORs with 95%CI adiposity exposure categories from WC, WHtR, C-Index, and LAP with prevalence of hypertension as the outcome were also represented with the reference category or lowest quartile. Receiver-operating characteristic (ROC) curves analysis was adopted for each adiposity index to assess the ability to correctly discriminate hypertension in both genders, independently divided for men and women. Overall, the diagnostic accuracy of the indicator was quantified using Area Under Curve (AUC). Values for each AUC can be between 0 and 1. A diagnostic test with an AUC value of 1 is perfectly accurate, and one with 0.5 has no discrimination power. The differences value of two areas under the curve (AUC) were analyzed by the algorithms model developed by Hanley and McNeil [21] The maximum value of the Youden index is used to determine the best cutoff value. Sensitivity and specificity along with the likelihood ratio, positive predictive value (PPV) and negative predictive value (NPV) for different cutoffs of indices in predicting hypertension were calculated with 95% CI. A confidence interval of 95% was adopted for all tests. A two-sided *p*-value <0.05 was considered statistically significant.

## 3. Results

As noted above, the total study sample included 15,842 participants with 7,506 being men (47.4%) and 8336 being women (52.6%) (Table 1). The mean age was 59.3±13.2. In general, the prevalence of hypertension among Thais middle-aged and elderly was given to 39.85% of men (95%CI: 38.74 to 40.96) and 38.59% of women (95%CI: 37.55 to 39.64). An age-related increase in the hypertension rate was observed. The prevalence of hypertension among adults aged 35 to 49 was 18.96% (95%CI: 17.81 to 20.12); for those aged 50 to 64, it was 40.00% (95%CI: 38.69 to 41.32); and for persons over 65 years old, it was 53.18% (95%CI: 51.93 to 54.44). Disregarding gender, Thai hypertensive adults were older; lived predominantly in urban areas; had higher body weight, HC, SBP, DBP, FBG; and showed worse lipid profiles (high triglycerides, LDL-C and low HDL-C [in women only]) than non-hypertensive adults. Individuals with high blood pressure showed increases in all adiposity indicators, particularly BMI, WC, WHR, WHtR, C-Index, BAI, LAP, CMI, VAI in comparison to those with normal blood pressure. In terms of a gender-specific perspective, among hypertensive adults, men were younger, having less BMI and HC and a lower level of both HDL-C and LDL-C, WHtR, BAI, LAP, and VAI than women who tended to have a higher SBP, DBP, WHR, and C-Index value. In addition, men appeared to be better educated, had higher smoking and alcohol usage, and were more physically active than women.

Table 2 presents the discriminating performance and AUC of different anthropometric and adiposity indicators for hypertension by gender. Among adult men, the most powerful ability to discriminate hypertension was for WHtR (AUC: 0.658; 95% CI: 0.646 to 0.671; sensitivity 57.4%, and specificity 66.8%), followed by WC (AUC: 0.651; 95% CI: 0.638–0.664), and WHR (AUC: 0.650; 95% CI: 0.637–0.662). The predicting ability of LAP was highest among non-traditional indicators (AUC: 0.632; 95%CI: 0.620–0.645), followed by CMI, and VAI showed the weakest discriminating ability with AUC at 0.555. Among adult women, LAP appeared to be the most reliable indicator in predicting hypertension (AUC: 0.646; 95% CI: 0.634–0.658; sensitivity 62.3%, and specificity 60.4%). Most notably, the AUC value of LAP was significantly higher than BMI, WC, WHR, BAI, and CI, except for WHtR (*p*-value = 0.228). Overall, in both genders, the top four indicators having the most power in discriminating hypertension were WHtR (AUC: 0.640; 95% CI: 0.631–0.649; sensitivity 57.6%, and specificity 63.2%), followed by LAP (AUC: 0.636); WC (AUC: 0.633); and C-Index (AUC: 0.630). BMI performed significantly worse to the top ranked AUC of 0.603, whereas BAI showed the weakest ability to discriminate the presence of hypertension with AUC at 0.578. This value was significantly lower compared with the remaining indicators. The influence of age on the association of the anthropometric indices was explored in stratified analyses (ages 35–49, 50–64, and over 65 years), and the results indicated that the overall trend did not vary substantially by age and sex (Table A1).

Based on the ROC analysis, in both genders, the best predicting performance was WC, WHtR, C-Index, and LAP, expressed by the highest AUC, was further analyzed. In quartile analysis (Table 3), there was a graded dose-response documented relationship of the WC, WHtR, C-Index, and LAP with the prevalence of hypertension among Thai middle-aged and elderly population. The frequency of high blood pressure in both genders increased progressively with the heightened WC, WHtR, C-Index, and LAP value (*p*-value for trend <0.001). When comparing between the top (4th quartile) versus bottom (1st quartile), the proportion of hypertension increased by 2.21-fold in LAP, 2.20- in WHtR, 2.05- in WC, and 2.04- in C-Index.

The value is expressed as a percentage (95% CI). The prevalence of hypertension demonstrated a significant trend with a stepwise increase across ascending quartiles of WC, WHtR, C-Index, and LAP. Differences were compared by using Chi-square.

Table 4 shows the results for the linear regression analysis. WC, WHtR, C-Index and LAP remained steady as strong and independent determinants with SBP and DBP in both genders (all *p*-value <0.001). WC exhibited the highest coefficients, followed by WHtR, LAP, and C-Index. Even after adjusting for various potential confounding variables (model 3), for each additional 1 SD increase in WC, WHtR, LAP, and C-Index, we observed a 5.09-, 4.58-, 4.17- and 2.78-mmHg increase in SBP, and a 3.07-, 2.47-, 2.51- and 1.73-mmHg increase in DBP (all *p*-value <0.001), respectively.

In the multivariable regression analysis expressed as quartiles, cut-off point, and SD incensement and continuous variable (Table 5), WC, WHtR, C-Index, and LAP consistently remained powerful and significantly associated with hypertension prevalence in all models. In the age-adjusted model (model 1), for each additional 1 SD increment in a particular adiposity index score, the odds ratio of hypertension were raised as highest from WC, OR: 1.776 [95% CI: 1.713 to 1.842], followed by WHtR, OR: 1.717 [95% CI: 1.657 to 1.780); LAP, OR: 1.648 [95% CI: 1.586 to 1.713]; and C-Index, OR: 1.442 [95% CI: 1.392 to 1.494], whereas they were slight reduced in the fully adjusted model (model 3) (WC, OR: 1.688 [95% CI: 1.623 to 1.756]; WHtR, OR: 1.629 [95% CI: 1.567 to 1.694]; LAP, OR: 1.602 [95% CI: 1.535 to 1.671]; C-Index, LAP, OR: 1.343 [95% CI: 1.293 to 1.394], all *p*-value <0.001). In quartiles regression analysis, the presence of hypertension increased steadily with the higher value of WC, WHtR, C-Index, and LAP quartiles. Particularly, in the most complex model (model 3), the increment of 425%, 374%, 352%, and 214% increased in odds ratio from the 25th percentile to the 75th percentile with LAP, WC, WHtR, and C-Index quartile, respectively. In the simplest model (model 1), the outcomes of higher OR were reported. The association between the abnormal anthropometric indices (defined by optimal cut-off point) and hypertension was analysed. In the third model, Thai aged over 35 with abnormal anthropometric indicators were approximately two to three times more likely to have hypertension than those at the normal cut-off. The best independent predictors of hypertension were the LAP (OR: 2.461 [95% CI: 2.277 to 2.660], followed by WC (OR: 2.360), WHtR (OR: 2.170), and C-Index (OR: 1.693).

## 4. Discussion

This study is the first to investigate the association of anthropometric and adiposity indices for hypertension among Thailand’s middle-aged and elderly population. Overall, the study revealed that all nine indices could be surrogate indicators to discriminate hypertension based on the findings of ROC analysis, although the differences were small, and with overlapping 95% CI. The top four indicators (WHtR, WC, C-index and LAP) with the best discriminating ability of hypertension were further analyzed by logistic regression. WHtR appeared to be the strongest predictor in both genders, followed by LAP, WC, and C-Index. All above indicators substantially exhibited a robust association with SBP and DBP in both genders. Multivariable logistic regression pinpointed a dose-response association of WC, WHtR, C-Index, and LAP with the prevalence of hypertension, which steadily increased and followed the higher quartiles in all adjusted model with a wide range of established confounder factors. These results highlight that these indices could be applied to assess body pattern linked to hypertension.

BMI with its single estimation of body composition does not distinguish between adipose tissue and lean mass [7]. Moreover, given that Asian and Western populations differ in their body-fat distribution, which may lead to false healthy-BMI and predisposition to visceral fat accumulation and insulin resistance, it is possible that BMI might not become a reasonable diagnostic parameter for detecting hypertension and cardio-metabolic risks [22,23]. The discrimination of fat stored in body compartments has been an attractive topic for various studies in order to clarify the influences of abdominal obesity on cardiometabolic disorders. Various clear-cut indicators, such as BAI or C-Index were discovered to meet the demands. The present study concluded that BAI and C-Index were likely to be inferior in predicting hypertension compared with other indicators, such as WHtR, WHR, WC or non-traditional index (LAP). These findings are similar with other results from previous studies [24,25,26,27,28], possibly due to its incompatible equation and significant differences in sample population with regard to age range, race, and ethnicity (based on the Caucasian population).

The simple assessment of WC has been designed to be an alternative for subcutaneous fat measurement [8]. In this study, WC showed exceptional discrimination and association with hypertension in both genders. The value of AUC was slightly lower than WHtR and LAP, however, it exhibited the best association with SBD, DBP, and presence of hypertension in logistic regression analyses. Nevertheless, some limitations of WC should be acknowledged, for instance, it does not express weight or height variables in its component, thus, potentially under- or over-estimating the risk, especially for short and tall individuals, respectively. Improved adiposity indicators were studied to overcome the disadvantages, such as WHtR or WHR [9]. The finding reveals that WHtR was the best predictor for discriminating the presence of hypertension, superior to WC, WHR, BAI, and C-Index and even to advanced invasive indicators such as LAP, CMI and VAI. Thus, using WHtR in hypertension prevention may be suitable. This finding is relevant to the outcomes from previous systematic review and meta-analysis studies on the association of anthropometric measures with the incidence of hypertension. These reviews and studies reported that the ability of WHtR in identifying hypertension was superior to other anthropometric indexes in both genders from various nationalities and ethnic groups [29,30,31,32]. Nevertheless, it is worth noting that the best adiposity proxies for diagnosis of hypertension remain controversial with incompatible conclusions. A case-control study conducted in 27,000 participants from 52 countries worldwide affirmed the predictive ability of WHR was more exceptional in detecting myocardial infarction than BMI [33], whereas, BMI, WC, and WHR were likely to be equivalently associated with hypertension and fluctuated in different age groups and genders in the Chinese population [34,35,36].

Despite the capability of WHtR or WHR and WC to measure adipose tissue distribution, several criticisms dismiss its application for monitoring, such as its vague biological interpretation; its greater variability across age, sex, race and ethnic groups; and its fluctuation in waist and hip values depending on the weight change [9]. Furthermore, there has not been a standardized accurate measurement of WC and HC and the calibration still faces challenges, especially in terms of being time-consuming and requiring assistance, particularly in obese or bed-ridden patients [37]. It is worth mentioning that these indices are functions of both the SAT and VAT compartments, while VAT represents a pathological adipose tissue depot and is more highly correlated with diabetes, hypertension, and adverse cardiovascular events. It is crucial to find a novel and more accurate clinical indicator for the determination of total body-fat distribution and visceral adipose tissue. Obesity is given attention as a heterogeneous condition and metabolic risk can fluctuate extensively from the differences in adipose tissue distribution [12,13,14]. Consequently, a better understanding of the link between hypertension and distinct adipose depots can facilitate the explanation of this variation.

Various complicated and non-traditional anthropometric indicators have been proposed to establish adiposity phenotypes and their associations with diseases such as CMI, VAI, and LAP. Nonetheless, there is an ongoing controversy as to which parameter of central obesity should be used in routine clinical practices or community setting to assess excess visceral deposition of total body fat-related hypertension. The present result reveals that LAP is statistically superior to BMI, WHR, BAI, and the complicated indicators such as CMI and VAI in order to discriminate hypertension. Similar findings also obtained from previous epidemiological studies among Chinese urban and rural habitants, Inner Mongolian, and Japanese adults suggested that LAP was positively and significantly associated with the risk of hypertension in both genders [38,39,40,41]. Conversely, LAP might not be superior to WHtR in predicting CVD in a 8.6-year follow-up prospective study among the Iranian population [42]. The reasons for inconsistent results may come from the component of LAP (based on WC and TG), which better reflects the fat-body accumulation and visceral fat, rather than simple height and body weight. Furthermore, LAP focuses on the independent relationship with metabolic syndrome and incident CVD events, that may support its high predicting hypertension compared with CMI and VAI [17]. Inversely, previous studies reported the measurement of central and general body-fat in predicting CVD has similar results, which explains the inferiority of LAP to WHtR in the present finding [43,44]. In this study, LAP is emerging as a beneficial index for health screening purposes with its simplicity and less components for estimating the lipid burden, compared to CMI and VAI, but inferior to WC or WHtR in hypertension prediction. This study also shows the robust associations of different indicators with hypertension, independent of the significant effect of confounders. In women, LAP, rather than BMI and WHtR, showed stronger prediction to hypertension, whereas WHtR was highly calibrated to LAP to detect high blood pressure in men. This finding may draw attention to the implied association of gender-specific body-fat phenotypes (both VAT and SAT) and LAP as the measurement tool for VAT. Women have more body fat with lower deleterious metabolic consequences than men, particularly characterized by a higher SAT amount, whereas they have lower VAT mass than men [45]. Consequently, any variation of VAT among women would be a more sensitive reflection of the LAP value. Considering the recent prospective association of WC, WHtR and LAP, the predictability of these tools might reflect the quantitative evaluation of total fat mass (defined by WC, WHtR) and assessing excess visceral deposition (defined by LAP) with hypertension.

Some potential limitations of this study should be acknowledged. First, we are unable to assign causality to our findings due to the nature of the cross-sectional study. Further experimental studies are needed to determine whether modification of the different types of adipose tissue (SAT or VAT) can result in improvement of the discriminating ability to hypertension. Second, hypertension was classified as a blood pressure measurement reading of 140/90 mmHg or use of blood pressure lowering medicine followed by JNC7. The blood pressure was read from a single day for measurement, which limited the precise estimation of the prevalence of hypertension. The present study analyzed the secondary data obtained from NHES-IV, which lacked a comprehensive evaluation of other confounding factors such as side-effects of medication consumption, sodium intake and the presence of diabetes. Third, given that our study was conducted among Thai middle-aged and elderly adults, it is uncertain whether the study’s findings can be generalized to other age groups and racial or ethnic populations.

Although our findings were concluded from an observational design, the results supported the healthy conception of keeping fit and lean based on the recommendation for the Asian population from the World Health Organization [46,47]. Managing BMI under 24.0; WC under 81.58; and WHtR under 0.52 in both genders may be the key for hypertension prevention among Thais aged over 35. Since the complicated, non-traditional (using anthropometrical and biochemical components), and invasive (using lipid profiles parameters) adiposity indicators for an exact determination of fat composition such as LAP, VAI or CMI are not easily available in large epidemiological studies, alternative simple, non-invasive and inexpensive measurements of general body fat distribution such as WHtR or WC could properly examine the association of adipose tissue with hypertension. Therefore, it could support in determining obesity-related hypertension burdens.

## 5. Conclusions

The comparison of nine adiposity indices provides an understanding of the association of hypertension with body fat variation among Thai adults aged over 35. Based on the analysis, we suggested the use of WHtR (>0.52) in both genders as the most simple, non-invasive and inexpensive index for estimating the association of body-fat with hypertension. Keeping fit and lean may be the best suggestion for the primary prevention of hypertension.

## Figures and Tables

**Table 1:** Anthropometric and biochemical characteristics of the study subjects.

Variable	Men		Women	
	Normotension (*N* = 4515)	Hypertension (*N* = 2991)	Normotension (*N* = 5119)	Hypertension (*N* = 3217)
**Age (years)**	57.1 ± 13.1	63.8 ± 11.9 ##	55.2 ± 13.2 **	64.4±11.5 ##*
**Living area**				
Rural	2316 (51.3)	1231 (41.2) ##	2337 (45.7) **	1337 (41.6) ##
Urban	2199 (48.7)	1760 (58.8) ##	2782 (54.3) **	1880 (58.4) ##
**Education background**				
Illiterate	167 (3.7)	134 (4.5) #	465 (9.1) **	441 (13.7) ##**
Primary school	3139 (69.7)	2036 (68.3) #	3514 (68.9) **	2366 (73.7) ##**
Secondary and vocational	951 (21.1)	677 (22.7) #	799 (15.7) **	332 (10.3) ##**
University and higher	248 (5.5)	133 (4.5) #	325 (6.3) **	71 (2.3) ##**
**Current smoking w/12 mo.**	2040 (45.2)	917 (30.7) ##	221 (4.3) **	120 (3.7) ##**
**Current regular smoking**	1794 (39.7)	766 (25.6) ##	169 (3.3) **	89 (2.8) ##**
**Alcohol drinking w/12 mo.**	2507 (55.6)	1437 (48.1) ##	1263 (24.7) **	464 (14.4) ##**
**Alcohol drinking level**				
Abstainer	2564 (56.8)	1881 (63.0) ##	4499 (88.0) **	2964 (92.2) ##**
Low risk	1475 (32.7)	752 (25.2) ##	436 (8.5) **	136 (4.2) ##**
Moderate risk	126 (2.8)	89 (3.0) ##	32 (0.6) **	21 (0.7) ##**
High risk	119 (2.6)	82 (2.7) ##	20 (0.4) **	7 (0.2) ##**
Severe risk	124 (2.7)	88 (2.9) ##	20 (0.4) **	16 (0.5) ##**
Drinking N/A amount	105 (2.4)	94 (3.2) ##	107 (2.1) **	71 (2.2) ##**
**Physical activity level**				
Low level	960 (21.5)	756 (25.5) ##	1153 (22.8) **	998 (31.5) ##**
Moderate level	1136 (25.5)	866 (29.3) ##	1526 (30.1) **	1019 (32.1) ##**
High level	2365 (53.0)	1337 (45.2) ##	2385 (47.1) **	1154 (36.4) ##**
**Height (cm)**	162.9 ± 6.3	162.7 ± 6.2	152.4 ± 5.8 **	151.2 ± 5.8 ##**
**Body Weight (kg)**	59.7 ± 11.5	64.0 ± 12.4 ##	56.1 ± 11.5 **	58.7 ± 12.7 ##**
**BMI (kg/m^2^)**	22.43 ± 3.74	24.11 ± 4.05 ##	24.07 ± 4.42 **	25.57 ± 4.95 ##**
**WC (cm)**	79.0 ± 10.5	84.9 ± 11.3 ##	78.5 ± 10.7 *	83.1 ± 11.6 ##**
**HC (cm)**	89.5 ± 7.5	92.3 ± 8.0 ##	93.4 ± 9.0 **	96.0 ± 10.3 ##**
**SBP (mmHg)**	119.2 ± 11.5	148.2 ± 19.9 ##	117.5 ± 12.0 **	145.7 ± 19.8 ##**
**DBP (mmHg)**	73.5 ± 8.4	86.5 ± 12.3 ##	71.5 ± 8.4 **	81.8 ± 11.6 ##**
**FBG (mg/dL)**	88.0 (81.0–97.0)	94.0 (85.0–107.0) ##	86.0 (80.0–95.0) **	93.0 (85.0–107.0) ##
**TG (mg/dL)**	129.4 (93.0–185.2)	142.6 (101.9–209.1) ##	116.9 (85.1–165.7) **	147.1 (106.3–201.1) ##
**HDL-C (mg/dL)**	43.2 (36.7–51.4)	43.2 (36.7–51.0)	47.9 (40.6–56.0) **	46.3 (39.2–54.1) ##**
**LDL-C (mg/dL)**	127.7 (101.9–153.6)	128.5 (102.8–157.0) ##	137.3 (112.3–163.7) **	141.7 (112.9-172.8) ##**
**WHR**	0.88 ± 0.07	0.92 ± 0.07 ##	0.84 ± 0.07 **	0.86 ± 0.07 ##**
**WHtR**	0.49 ± 0.06	0.52 ± 0.07 ##	0.52 ± 0.07 **	0.55 ± 0.08 ##**
**C-Index**	1.20 ± 0.08	1.24 ± 0.08 ##	1.19 ± 0.09 **	1.23 ± 0.10 ##**
**BAI**	25.08 ± 3.52	26.54 ± 3.74 ##	31.69 ± 4.84 **	33.68 ± 5.46 ##**
**LAP**	18.21 (7.35–38.28)	31.03 (15.54–56.12) ##	25.61 (13.80–44.26) **	39.74 (23.25–65.24) ##**
**CMI**	1.44 (0.90–2.42)	1.74 (1.05–2.93) ##	1.25 (0.78–2.04) **	1.74 (1.11–2.73) ##
**VAI**	1.60 (1.02–2.61)	1.83 (1.14–3.05) ##	1.91 (1.22–3.10) **	2.54 (1.65–3.93) ##**

Value is expressed as mean ± standard deviation or median (interquartile range) or numbers (percentage) as appropriate. The asterisk denotes significant differences between normotension and hypertension subjects within the same gender (# *p* < 0.05, ## *p* < 0.001) and differences between men and women in the same disease group (* *p* < 0.05, ** *p* < 0.001). Differences were compared by Independent T-test or Mann–Whitney U test or Chi-square test appropriately.

**Table 2:** Area under curve (AUC), cut-off point and diagnostic performance of various anthropometric and adiposity indices with hypertension classified by gender.

Indicator	AUC (95%CI)	Cutoff	J	Sensitivity	Specificity	Likelihood Ratio	PPV	NPV
**Men**								
Body mass index	0.624 (0.611–0.637)	23.02	0.185	59.3 (57.5–61.0)	59.2 (57.7–60.6)	1.45 (1.39–1.52)	49.0 (47.4–50.7)	68.7 (67.2–70.1)
Waist circumference	0.651 (0.638–0.664) ^a^	81.42	0.230	62.0 (60.2–63.7)	61.0 (59.6–62.4)	1.59 (1.52–1.67)	51.3 (49.7–52.9)	70.8 (69.3–72.2)
Waist-to-hip ratio	0.650 (0.637–0.662) ^a^	0.91	0.233	55.4 (53.6–57.2)	67.6 (66.2–69.0)	1.71 (1.62–1.80)	53.1 (51.3–54.8)	69.6 (68.2–70.9)
Waist-to-height ratio	0.658 (0.646–0.671) ^a^	0.51	0.243	57.4 (55.6–59.2)	66.8 (65.4–68.2)	1.73 (1.64–1.82)	53.4 (51.7–55.1)	70.3 (68.9–71.7)
Conicity index	0.649 (0.636–0.662) ^a^	1.22	0.228	62.4 (60.6–64.1)	59.9 (58.4–61.3)	1.56 (1.49–1.63)	50.7 (49.1–52.4)	70.6 (69.1–72.1)
Body adiposity index	0.614 (0.601–0.627) ^a–e^	25.34	0.167	61.3 (59.5–63.1)	55.3 (53.8–56.8)	1.37 (1.31–1.43)	47.6 (46.0–49.2)	68.3 (66.8–69.8)
Lipid accumulation product	0.632 (0.620–0.645) ^b,d,f^	21.48	0.213	65.8 (64.1–67.4)	55.5 (54.0–57.0)	1.48 (1.42–1.54)	49.5 (47.9–51.1)	71.0 (69.5–72.5)
Cardiometabolic index	0.569 (0.556–0.582) ^a–g^	1.69	0.108	51.5 (49.7–53.3)	59.1 (57.6–60.5)	1.26 (1.20–1.32)	45.5 (43.8–47.2)	64.8 (63.3–66.2)
Visceral adiposity index	0.555 (0.542–0.569) ^a–g^	1.94	0.086	46.8 (45.0–48.6)	61.6 (60.2–63.0)	1.22 (1.16–1.29)	44.7 (43.0–46.5)	63.6 (62.2–65.1)
**Women**								
Body mass index	0.591 (0.579–0.604)	24.78	0.146	54.6 (52.9–56.4)	59.9 (58.5–61.2)	1.36 (1.30–1.43)	46.1 (44.5–47.7)	67.8 (66.4–69.1)
Waist circumference	0.615 (0.603–0.628) ^a^	81.53	0.182	54.4 (52.7–56.1)	63.8 (62.5–65.2)	1.50 (1.43–1.58)	48.6 (47.0–50.2)	69.0 (67.7–70.3)
Waist-to-hip ratio	0.605 (0.593–0.618)	0.84	0.159	63.4 (61.7–65.1)	52.4 (51.0–53.7)	1.33 (1.28–1.38)	45.5 (44.1–47.0)	69.5 (68.0–70.9)
Waist-to-height ratio	0.632 (0.620–0.644) ^a,c^	0.54	0.196	53.2 (51.5–55.0)	66.1 (64.8–67.4)	1.57 (1.50–1.65)	49.7 (48.0–51.4)	69.2 (67.9–70.5)
Conicity index	0.614 (0.601–0.626) ^d^	1.21	0.170	56.3 (54.5–58.0)	60.4 (59.0–61.7)	1.42 (1.36–1.48)	47.2 (45.6–48.8)	68.7 (67.3–70.1)
Body adiposity index	0.607 (0.595–0.620) ^d^	34.19	0.164	42.7 (41.0–44.5)	73.7 (72.5–74.9)	1.62 (1.53–1.73)	50.5 (48.6–52.4)	67.2 (65.9–68.4)
Lipid accumulation product	0.646 (0.634–0.658) ^a–c,e,f^	31.59	0.227	62.3 (60.6–63.9)	60.4 (59.1–61.8)	1.57 (1.51–1.64)	49.7 (48.2–51.3)	71.8 (70.4–73.1)
Cardiometabolic index	0.630 (0.618–0.642) ^a,c,e,g^	1.23	0.194	70.0 (68.4–71.6)	49.3 (48.0–50.7)	1.38 (1.33–1.43)	46.5 (45.1–47.9)	72.4 (70.9–73.9)
Visceral adiposity index	0.618 (0.606–0.630) ^d,g^	1.74	0.173	72.5 (70.9–74.1)	44.7 (43.3–46.0)	1.31 (1.27–1.35)	45.2 (43.8–46.5)	72.1 (70.5–73.7)
**Both gender**								
Body mass index	0.603 (0.594–0.612)	23.97	0.155	55.2 (54.0–56.5)	60.2 (59.3–61.2)	1.39 (1.34–1.44)	47.2 (46.1–48.4)	67.6 (66.6–68.6)
Waist circumference	0.633 (0.624–0.641) ^a^	81.58	0.205	57.7 (56.5–59.0)	62.8 (61.8–63.8)	1.55 (1.50–1.60)	50.0 (48.8–51.2)	69.7 (68.8–70.7)
Waist-to-hip ratio	0.620 (0.611–0.629) ^a^	0.87	0.174	60.1 (58.8–61.3)	57.2 (56.2–58.2)	1.40 (1.36–1.45)	47.5 (46.4–48.6)	69.0 (67.9–70.0)
Waist-to-height ratio	0.640 (0.631–0.649) ^a,c^	0.52	0.210	57.6 (56.4–58.9)	63.2 (62.2–64.1)	1.56 (1.51–1.62)	50.2 (49.0–51.4)	69.8 (68.8–70.8)
Conicity index	0.630 (0.621–0.639) ^a^	1.21	0.197	61.3 (60.0–62.5)	58.1 (57.1–59.1)	1.46 (1.42–1.51)	48.5 (474–49.6)	70.0 (68.9–70.1)
Body adiposity index	0.578 (0.569–0.587) ^a–e^	27.53	0.102	64.1 (62.9–65.3)	46.0 (45.0–47.0)	1.19 (1.16–1.22)	43.4 (42.4–44.4)	66.6 (65.4–67.7)
Lipid accumulation product	0.636 (0.627–0.645) ^a,c,f^	24.44	0.208	67.3 (66.1–68.4)	53.6 (52.6–54.6)	1.45 (1.41–1.49)	48.3 (47.2–49.3)	71.7 (70.7–72.8)
Cardiometabolic index	0.601 (0.592–0.610) ^b–g^	1.55	0.149	56.5 (55.2–57.7)	58.4 (57.4–59.3)	1.36 (1.32–1.40)	46.6 (45.5–47.8)	67.5 (66.5–68.5)
Visceral adiposity index	0.586 (0.577–0.595) ^a–e,g,h^	1.73	0.127	63.5 (62.3–64.7)	49.2 (48.2–50.2)	1.25 (1.22–1.28)	44.6 (43.6–45.6)	67.6 (66.5–68.7)

Values in parentheses are 95% confidence intervals (CI). Abbreviation: AUC Area under curve; J Youden index; PPV positive predictive value, NPV negative predictive value. ^a^ indicates a significant difference as compared to BMI; ^d^ indicates a significant difference as compared to WHtR; ^g^ indicates a significant difference as compared to LAP; ^b^ indicates a significant difference as compared to WC; ^e^ indicates a significant difference as compared to C-Index; ^h^ indicates a significant difference as compared to CMI; ^c^ indicates a significant difference as compared to WHR; ^f^ indicates a significant difference as compared to BAI.

**Table 3:** The prevalence of hypertension by quartiles of WC, WHtR, C-Index, and LAP in both genders.

	Quartile 1	Quartile 2	Quartile 3	Quartile 4	p For Trend
WC	26.88 (25.50–28.26)	32.82 (31.36–34.28)	42.29 (40.75–43.82)	55.08 (53.51–56.64)	<0.001
WHtR	24.91 (23.56–26.26)	33.74 (32.26–35.21)	43.24 (41.70–44.79)	54.86 (53.31–56.41)	<0.001
C-Index	26.75 (25.37–28.13)	33.26 (31.79–34.73)	42.21 (40.67–43.75)	54.53 (52.98–56.08)	<0.001
LAP	24.29 (22.96–25.63)	34.60 (33.11–36.08)	44.09 (42.55–45.64)	53.76 (52.21–55.32)	<0.001

**Table 4:** Linear regression models predicting change (95% CI) in blood pressure (SBP and DBP) per SD incensement in WC, WHtR, C-Index, and LAP in both-genders.

Variables	SBP (mmHg)			DBP (mmHg)		
	*B* (95% CI)	β	*p*-value	*B* (95% CI)	β	*p*-value
WC						
Model 1	5.391 (5.094–5.689)	0.258	<0.001	3.147 (2.974–3.320)	0.273	<0.001
Model 2	5.093 (4.774–5.412)	0.244	<0.001	3.073 (2.888–3.258)	0.266	<0.001
WHtR						
Model 1	4.920 (4.620–5.219)	0.236	<0.001	2.506 (2.331–2.681)	0.217	<0.001
Model 2	4.581 (4.260–4.903)	0.220	<0.001	2.465 (2.278–2.653)	0.214	<0.001
C-Index						
Model 1	3.342 (3.030–3.654)	0.160	<0.001	1.987 (1.806–2.167)	0.172	<0.001
Model 2	2.784 (2.461–3.107)	0.134	<0.001	1.727 (1.539–1.914)	0.150	<0.001
LAP						
Model 1	4.227 (3.925–4.529)	0.203	<0.001	2.411 (2.236–2.587)	0.209	<0.001
Model 2	4.169 (3.842–4.496)	0.200	<0.001	2.514 (2.324–2.704)	0.218	<0.001

Models constructed with dependent variables as SBP and DBP, and independent variables as WC, WHtR, C-Index, and LAP. Values in table are unstandardized (B) with 95% CI, standardized correlation coefficients (β), and p-values. Unstandardized (B) and standardized (β) coefficients are per 1 SD increment of WC, WHtR, C-Index, and LAP. Model 1: age-adjusted; Model 2: further adjusted for living area, education background, cigarette smoking within 12 months and regular smoking, alcohol consumption, alcohol consumption level, physical activity, fasting plasma glucose*, HDL-C*and LDL-C*. *: Variable is for the log. 95% CI: 95% confidence interval.

**Table 5:** Multivariate logistic regression of WC, WHtR, C-Index, and LAP for presence of hypertension in both genders.

Variable	Odds Ratio (95% CI)					
	Model 1	*p*	Model 2	*p*	Model 3	*p*
*Quartiles of WC*						
Q1 (≤72.75)	1.000 (Ref)	–	1.000 (Ref)	–	1.000 (Ref)	–
Q2 (72.75–80.25)	1.594 (1.439–1.765)	<0.001	1.557 (1.404–1.728)	<0.001	1.527 (1.375–1.697)	<0.001
Q3 (80.25–88.00)	2.484 (2.247–2.747)	<0.001	2.392 (2.158–2.650)	<0.001	2.289 (2.060–2.544)	<0.001
Q4 (>88.00)	4.317 (3.900–4.779)	<0.001	4.038 (3.638–4.482)	<0.001	3.742 (3.355–4.174)	<0.001
*p* for trend		<0.001		<0.001		<0.001
WC (per 1SD increment)	1.776 (1.713–1.842)	<0.001	1.736 (1.673–1.802)	<0.001	1.688 (1.623–1.756)	<0.001
WC (cut–off point >81.58)	2.636 (2.459–2.826)	<0.001	2.511 (2.339–2.696)	<0.001	2.360 (2.191–2.542)	<0.001
*Quartiles of WHtR*						
Q1 (≤0.46)	1.000 (Ref)	–	1.000 (Ref)	–	1.000 (Ref)	–
Q2 (0.46–0.51)	1.678 (1.515–1.860)	<0.001	1.641 (1.478–1.822)	<0.001	1.616 (1.453–1.797)	<0.001
Q3 (0.51–0.56)	2.547 (2.303–2.817)	<0.001	2.455 (2.213–2.724)	<0.001	2.343 (2.105–2.607)	<0.001
Q4 (>0.56)	4.025 (3.640–4.451)	<0.001	3.838 (3.457–4.262)	<0.001	3.525 (3.162–3.931)	<0.001
*p* for trend		<0.001		<0.001		<0.001
WHtR (per 1SD increment)	1.717 (1.657–1.780)	<0.001	1.688 (1.626–1.753)	<0.001	1.629 (1.567–1.694)	<0.001
WHtR (cut–off point >0.52)	2.445 (2.283–2.619)	<0.001	2.332 (2.172–2.504)	<0.001	2.170 (2.016–2.336)	<0.001
*Quartiles of Conicity Index*						
Q1 (≤1.15)	1.000 (Ref)	–	1.000 (Ref)	–	1.000 (Ref)	–
Q2 (1.15–1.21)	1.320 (1.194–1.460)	<0.001	1.291 (1.166–1.429)	<0.001	1.251 (1.129–1.387)	<0.001
Q3 (1.21–1.27)	1.900 (1.722–2.095)	<0.001	1.822 (1.649–2.013)	<0.001	1.705 (1.540–1.888)	<0.001
Q4 (>1.27)	2.591 (2.349–2.858)	<0.001	2.435 (2.204–2.691)	<0.001	2.140 (1.929–2.373)	<0.001
*p* for trend		<0.001		<0.001		<0.001
C–Index (per 1SD increment)	1.442 (1.392–1.494)	<0.001	1.411 (1.360–1.462)	<0.001	1.343 (1.293–1.394)	<0.001
C–Index (cut–off point >1.21)	1.925 (1.799–2.061)	<0.001	1.847 (1.723–1.979)	<0.001	1.693 (1.576–1.818)	<0.001
*Quartiles of LAP*						
Q1 (≤13.04)	1.000 (Ref)	–	1.000 (Ref)	–	1.000 (Ref)	–
Q2 (13.04–27.21)	1.857 (1.675–2.059)	<0.001	1.777 (1.599–1.974)	<0.001	1.804 (1.621–2.008)	<0.001
Q3 (27.21–49.59)	2.788 (2.518–3.086)	<0.001	2.648 (2.386–2.938)	<0.001	2.704 (2.425–3.015)	<0.001
Q4 (>49.59)	4.431 (4.001–4.908)	<0.001	4.186 (3.770–4.649)	<0.001	4.251 (3.792–4.765)	<0.001
*p* for trend		<0.001		<0.001		<0.001
LAP (per 1SD increment)	1.648 (1.586–1.713)	<0.001	1.616 (1.554–1.680)	<0.001	1.602 (1.535–1.671)	<0.001
LAP (cut–off point >24.44)	2.631 (2.451–2.823)	<0.001	2.526 (2.350–2.716)	<0.001	2.461 (2.277–2.660)	<0.001

Model 1: Age-adjusted; Model 2: further adjusted for living area, education background, cigarette smoking within 12 months and regular smoking, alcohol consumption, alcohol consumption level, physical activity; Model 3: further adjusted for fasting plasma glucose*, HDL-C*, LDL-C*. *: Variable is for the log. OR: odds ratio; 95% CI: 95% confidence interval.

## Appendix A

**Table A1:** Area under curve (AUC), cut-off point and diagnostic performance of various anthropometric and adiposity indices with hypertension classified by age and gender.

Indicator	35–49 Years Old	50–64 Years Old	Over 65 Years Old
AUC (95% CI)			
**Men**			
Body mass index	0.653 (0.622–0.683)	0.674 (0.652–0.695)	0.654 (0.635–0.674)
Waist circumference	0.660 (0.630–0.689)	0.683 (0.662–0.704)	0.658 (0.638–0.677)
Waist-to-hip ratio	0.652 (0.622–0.681)	0.663 (0.641–0.684)	0.623 (0.604–0.643)
Waist-to-height ratio	0.662 (0.633–0.692)	0.680 (0.659–0.701)	0.651 (0.631–0.670)
Conicity index	0.637 (0.607–0.667)	0.646 (0.624–0.668)	0.617 (0.597–0.637)
Body adiposity index	0.618 (0.587–0.648)	0.628 (0.606–0.650)	0.617 (0.597–0.637)
Lipid accumulation product	0.660 (0.631–0.689)	0.665 (0.644–0.687)	0.647 (0.628–0.667)
Cardiometabolic index	0.607 (0.577–0.637)	0.582 (0.559–0.604)	0.572 (0.552–0.592)
Visceral adiposity index	0.594 (0.564–0.625)	0.564 (0.542–0.587)	0.557 (0.537–0.578)
**Women**			
Body mass index	0.685 (0.657–0.712)	0.638 (0.617–0.658)	0.616 (0.597–0.636)
Waist circumference	0.689 (0.662–0.717)	0.632 (0.612–0.653)	0.608 (0.588–0.628)
Waist-to-hip ratio	0.649 (0.620–0.677)	0.590 (0.569–0.612)	0.568 (0.547–0.588)
Waist-to-height ratio	0.686 (0.659–0.714)	0.635 (0.614–0.656)	0.610 (0.590–0.630)
Conicity index	0.624 (0.594–0.654)	0.588 (0.567–0.610)	0.556 (0.535–0.576)
Body adiposity index	0.640 (0.610–0.670)	0.616 (0.595–0.637)	0.599 (0.579–0.619)
Lipid accumulation product	0.707 (0.681–0.733)	0.646 (0.625–0.666)	0.609 (0.589–0.629)
Cardiometabolic index	0.668 (0.641–0.695)	0.605 (0.584–0.626)	0.570 (0.549–0.590)
Visceral adiposity index	0.650 (0.622–0.677)	0.589 (0.568–0.610)	0.551 (0.531–0.572)
**Both gender**			
Body mass index	0.657 (0.636–0.678)	0.651 (0.636–0.666)	0.636 (0.622–0.650)
Waist circumference	0.677 (0.656–0.697)	0.656 (0.642–0.671)	0.632 (0.618–0.645)
Waist-to-hip ratio	0.657 (0.637–0.677)	0.612 (0.597–0.627)	0.585 (0.570–0.599)
Waist-to-height ratio	0.661 (0.640–0.681)	0.653 (0.638–0.667)	0.632 (0.618–0.645)
Conicity index	0.634 (0.614–0.655)	0.614 (0.599–0.629)	0.584 (0.570–0.599)
Body adiposity index	0.560 (0.538–0.582)	0.593 (0.578–0.609)	0.597 (0.583–0.611)
Lipid accumulation product	0.681 (0.661–0.701)	0.653 (0.638–0.668)	0.630 (0.616–0.644)
Cardiometabolic index	0.645 (0.625–0.665)	0.594 (0.578–0.609)	0.572 (0.558–0.587)
Visceral adiposity index	0.623 (0.602–0.644)	0.577 (0.562–0.593)	0.559 (0.545–0.574)

Values in parentheses are 95 % confidence intervals (CI). Abbreviation: AUC Area under curve.

## References

[1] Lim L.-Y.L., Kjellstrom T., Sleigh A., Khamman S., Seubsman S.-A., Dixon J., Banwell C. (2009). Associations between urbanisation and components of the health-risk transition in Thailand. A descriptive study of 87,000 Thai adults. Glob. Health Action.

[2] Juonala M., Magnussen C.G., Berenson G.S., Venn A., Burns T.L., Sabin M.A., Srinivasan S.R., Daniels S.R., Davis P.H., Chen W. (2011). Childhood adiposity, adult adiposity, and cardiovascular risk factors. N. Engl. J. Med..

[3] Leelacharas S. (2009). Hypertension in Thailand. Prog. Cardiovasc. Nurs..

[4] Kishore J., Gupta N., Kohli C., Kumar N. Prevalence of Hypertension and Determination of Its Risk Factors in Rural Delhi. https://www.hindawi.com/journals/ijhy/2016/7962595/.

[5] Mozaffarian D., Benjamin E.J., Go A.S., Arnett D.K., Blaha M.J., Cushman M., de Ferranti S., Després J.-P., Fullerton H.J., Howard V.J. (2015). Heart disease and stroke statistics—2015 update: A report from the American Heart Association. Circulation.

[6] Aekplakorn W., Mo-Suwan L. (2009). Prevalence of obesity in Thailand. Obes. Rev..

[7] Shuster A., Patlas M., Pinthus J.H., Mourtzakis M. (2012). The clinical importance of visceral adiposity: A critical review of methods for visceral adipose tissue analysis. Br. J. Radiol..

[8] Cornier M.-A., Després J.-P., Davis N., Grossniklaus D.A., Klein S., Lamarche B., Lopez-Jimenez F., Rao G., St-Onge M.-P., Towfighi A. (2011). Assessing adiposity: A scientific statement from the American Heart Association. Circulation.

[9] Browning L.M., Hsieh S.D., Ashwell M. (2010). A systematic review of waist-to-height ratio as a screening tool for the prediction of cardiovascular disease and diabetes: 0·5 could be a suitable global boundary value. Nutr. Res. Rev..

[10] Valdez R. (1991). A simple model-based index of abdominal adiposity. J. Clin. Epidemiol..

[11] Bergman R.N., Stefanovski D., Buchanan T.A., Sumner A.E., Reynolds J.C., Sebring N.G., Xiang A.H., Watanabe R.M. (2011). A better index of body adiposity. Obesity.

[12] Wajchenberg B.L., Giannella-Neto D., da Silva M.E., Santos R.F. (2002). Depot-specific hormonal characteristics of subcutaneous and visceral adipose tissue and their relation to the metabolic syndrome. Horm. Metab. Res..

[13] Tang L., Zhang F., Tong N. (2016). The association of visceral adipose tissue and subcutaneous adipose tissue with metabolic risk factors in a large population of Chinese adults. Clin. Endocrinol..

[14] Koh H., Hayashi T., Sato K.K., Harita N., Maeda I., Nishizawa Y., Endo G., Fujimoto W.Y., Boyko E.J., Hikita Y. (2011). Visceral adiposity, not abdominal subcutaneous fat area, is associated with high blood pressure in Japanese men: The Ohtori study. Hypertens. Res..

[15] Amato M.C., Giordano C. (2014). Visceral Adiposity Index: An Indicator of Adipose Tissue Dysfunction. Int. J. Endocrinol..

[16] Amato M.C., Giordano C., Galia M., Criscimanna A., Vitabile S., Midiri M., Galluzzo A., AlkaMeSy Study Group (2010). Visceral Adiposity Index: A reliable indicator of visceral fat function associated with cardiometabolic risk. Diabetes Care.

[17] Kahn H.S. (2005). The “lipid accumulation product” performs better than the body mass index for recognizing cardiovascular risk: A population-based comparison. BMC Cardiovasc. Disord..

[18] Wakabayashi I., Daimon T. (2015). The “cardiometabolic index” as a new marker determined by adiposity and blood lipids for discrimination of diabetes mellitus. Clin. Chim. Acta.

[19] Aekplakorn W., Chariyalertsak S., Kessomboon P., Sangthong R., Inthawong R., Putwatana P., Taneepanichskul S. (2011). Prevalence and Management of Diabetes and Metabolic Risk Factors in Thai Adults. Diabetes Care.

[20] Chobanian A.V., Bakris G.L., Black H.R., Cushman W.C., Green L.A., Izzo J.L., Jones D.W., Materson B.J., Oparil S., Wright J.T. (2003). The Seventh Report of the Joint National Committee on Prevention, Detection, Evaluation, and Treatment of High Blood Pressure: The JNC 7 report. JAMA.

[21] Hanley J.A., McNeil B.J. (1982). The meaning and use of the area under a receiver operating characteristic (ROC) curve. Radiology.

[22] Deurenberg P., Deurenberg-Yap M., Guricci S. (2002). Asians are different from Caucasians and from each other in their body mass index/body fat per cent relationship. Obes. Rev..

[23] Nazare J.-A., Smith J.D., Borel A.-L., Haffner S.M., Balkau B., Ross R., Massien C., Alméras N., Després J.-P. (2012). Ethnic influences on the relations between abdominal subcutaneous and visceral adiposity, liver fat, and cardiometabolic risk profile: The International Study of Prediction of Intra-Abdominal Adiposity and Its Relationship with Cardiometabolic Risk/Intra-Abdominal Adiposity. Am. J. Clin. Nutr..

[24] Melmer A., Lamina C., Tschoner A., Ress C., Kaser S., Laimer M., Sandhofer A., Paulweber B., Ebenbichler C.F. (2013). Body adiposity index and other indexes of body composition in the SAPHIR study: Association with cardiovascular risk factors. Obesity.

[25] Moliner-Urdiales D., Artero E.G., Sui X., España-Romero V., Lee D., Blair S.N. (2014). Body adiposity index and incident hypertension: The Aerobics Center Longitudinal Study. Nutr. Metab. Cardiovasc. Dis..

[26] de Oliveira C.M., Ulbrich A.Z., Neves F.S., Dias F.A.L., Horimoto A.R.V.R., Krieger J.E., de Oliveira Alvim R., da Costa Pereira A. (2017). Association between anthropometric indicators of adiposity and hypertension in a Brazilian population: Baependi Heart Study. PLoS ONE.

[27] Zhou Z., Hu D., Chen J. (2009). Association between obesity indices and blood pressure or hypertension: Which index is the best?. Public Health Nutr..

[28] Farzad S., Fatemeh A., Salehi M., Nojomi M. (2012). Association of waist circumference, body mass index and conicity index with cardiovascular risk factors in postmenopausal women. Cardiovasc. J. Afr..

[29] Lee C.M.Y., Huxley R.R., Wildman R.P., Woodward M. (2008). Indices of abdominal obesity are better discriminators of cardiovascular risk factors than BMI: A meta-analysis. J. Clin. Epidemiol..

[30] Ashwell M., Gunn P., Gibson S. (2012). Waist-to-height ratio is a better screening tool than waist circumference and BMI for adult cardiometabolic risk factors: Systematic review and meta-analysis. Obes. Rev..

[31] Jayedi A., Rashidy-Pour A., Khorshidi M., Shab-Bidar S. (2018). Body mass index, abdominal adiposity, weight gain and risk of developing hypertension: A systematic review and dose-response meta-analysis of more than 2.3 million participants. Obes. Rev..

[32] Lichtenauer M., Wheatley S.D., Martyn-St James M., Duncan M.J., Cobayashi F., Berg G., Musso C., Graffigna M., Soutelo J., Bovet P. (2018). Efficacy of anthropometric measures for identifying cardiovascular disease risk in adolescents: Review and meta-analysis. Minerva Pediatr..

[33] Yusuf S., Hawken S., Ounpuu S., Bautista L., Franzosi M.G., Commerford P., Lang C.C., Rumboldt Z., Onen C.L., Lisheng L. (2005). Obesity and the risk of myocardial infarction in 27,000 participants from 52 countries: A case-control study. Lancet.

[34] Deng W.-W., Wang J., Liu M.-M., Wang D., Zhao Y., Liu Y.-Q., Wang H., Dong G.-H. (2013). Body mass index compared with abdominal obesity indicators in relation to prehypertension and hypertension in adults: The CHPSNE study. Am. J. Hypertens..

[35] Lin S., Cheng T.O., Liu X., Mai J., Rao X., Gao X., Deng H., Shi M. (2006). Impact of dysglycemia, body mass index, and waist-to-hip ratio on the prevalence of systemic hypertension in a lean Chinese population. Am. J. Cardiol..

[36] Wu X., Li B., Lin W.-Q., Huang L.-L., Wang X.-X., Fu L.-Y., Li B.-B., Wang P.-X. (2018). The association between obesity indices and hypertension: Which index is the most notable indicator of hypertension in different age groups stratified by sex?. Clin. Exp. Hypertens..

[37] Molarius A., Seidell J.C. (1998). Selection of anthropometric indicators for classification of abdominal fatness—A critical review. Int. J. Obes. Relat. Metab. Disord..

[38] Zhong C., Xia W., Zhong X., Xu T., Li H., Zhang M., Wang A., Xu T., Sun Y., Zhang Y. (2016). Lipid Accumulation Product and Hypertension Related to Stroke: A 9.2-Year Prospective Study Among Mongolians in China. J. Atheroscler. Thromb..

[39] Shen Y.Y., Chen J.C., Li G., Cao J., Li J.X., Huang J.F., Gu D.F. (2017). Relationship of lipid accumulation product with hypertension and diabetes among Beijing residents study. Zhonghua Yu Fang Yi Xue Za Zhi.

[40] Song J., Zhao Y., Nie S., Chen X., Wu X., Mi J. (2018). The effect of lipid accumulation product and its interaction with other factors on hypertension risk in Chinese Han population: A cross-sectional study. PLoS ONE.

[41] Wakabayashi I., Daimon T. (2014). A strong association between lipid accumulation product and diabetes mellitus in japanese women and men. J. Atheroscler. Thromb..

[42] Bozorgmanesh M., Hadaegh F., Azizi F. (2010). Predictive performances of lipid accumulation product vs. adiposity measures for cardiovascular diseases and all-cause mortality, 8.6-year follow-up: Tehran lipid and glucose study. Lipids Health Dis..

[43] Auyeung T.W., Lee J.S.W., Leung J., Kwok T., Leung P.C., Woo J. (2010). Survival in older men may benefit from being slightly overweight and centrally obese—A 5-year follow-up study in 4000 older adults using DXA. J. Gerontol. A Biol. Sci. Med. Sci..

[44] Pischon T., Boeing H., Hoffmann K., Bergmann M., Schulze M.B., Overvad K., van der Schouw Y.T., Spencer E., Moons K.G.M., Tjønneland A. (2008). General and abdominal adiposity and risk of death in Europe. N. Engl. J. Med..

[45] Karastergiou K., Smith S.R., Greenberg A.S., Fried S.K. (2012). Sex differences in human adipose tissues—The biology of pear shape. Biol. Sex Differ..

[46] WHO Expert Consultation (2004). Appropriate body-mass index for Asian populations and its implications for policy and intervention strategies. Lancet.

[47] World Health Organization (2008). Waist Circumference and Waist-Hip Ratio: Report of a WHO Expert Consultation, Geneva, 8–11 December 2008.

